# Yiqi Yangyin Huoxue Method in Treating Diabetic Retinopathy: A Systematic Review and Meta-Analysis

**DOI:** 10.1155/2019/6020846

**Published:** 2019-04-02

**Authors:** Chen Ou, Yi Jing Yang, Qing Hua Peng

**Affiliations:** Hunan University of Chinese Medicine, Changsha, Hunan 410208, China

## Abstract

**Objective:**

To evaluate the efficacy of Yiqi Yangyin Huoxue method in the treatment of diabetic retinopathy (DR) with meta-analysis.

**Method:**

A randomized controlled trial of Yiqi Yangyin Huoxue method in the treatment of diabetic retinopathy in PubMed, Medline, Cochrane Library, Weipu Journal, China Knowledge Network, and Wanfang database was conducted. Two reviewers independently extracted data and methodological quality assessment. Data analysis was performed using Rev Man 5.3 software for statistical analysis.

**Results:**

A total of 10 randomized controlled trials, including 661 patients, were included. The results showed that Yiqi Yangyin Huoxue method could significantly improve the vision [risk ratio (RR)=1.32, 95% confidence interval (CI) (1.18, 1.47), P<0.00001] and change the eye fundus [RR=1.23, 95% CI (1.10, 1.37), P=0.0002], fundus fluorescence angiography (FFA) [RR=1.33, 95% CI (1.11, 1.60), P=0.002], traditional Chinese medicine syndromes [RR=1.31, 95% CI (1.15, 1.49), P<0.0001], and hemorheological parameters [mean difference (MD) =-0.37, 95% CI (-0.41, -0.32), P<0.00001].

**Conclusion:**

Yiqi Yangyin Huoxue method showed beneficial effects for DR on improving vision, eye fundus, FFA, TCM syndromes, and hemorheological parameters.

## 1. Introduction

Diabetic retinopathy (DR) is one of the most common and serious complications of diabetes mellitus, which remains the leading cause of vision loss in adults worldwide [1, 2]. A recent systematic review of 35 population-based studies around the world showed that the prevalence of DR among people with diabetes was 34.6% [3]. Worldwide, there are about 93 million people with diabetic retinopathy, including 21 million people with diabetic macular edema and 17 million people with proliferative diabetic retinopathy [4].

Traditional Chinese medicine (TCM) is a style of traditional medicine built on a distinct foundation of more than at least 2500 years of Chinese medical practice and recently influenced by modern Western medicine. Ancient Chinese scholars noted that all natural phenomena could be categorized into Yin and Yang (two opposite, complementary, interdependent, and exchangeable aspects of nature). Yin refers largely to the material aspects of the organism and Yang to functions. The organs work together by regulating and preserving qi (energy) and blood through the so-called channels and collaterals [5]. TCM considers that DR is a “diabetes cataract”. Early eyes often have no obvious symptoms. In the middle and late stage, it can cause opacity of the lens, fundus hemorrhage, edema, exudation, neovascularization, and other intraocular lesions [6]. Nowadays, TCM is widely used in the treatment of DR. The main basis of treatment in TCM is syndrome differentiation. According to syndrome differentiation, deficiency of qi and yin and blood stasis is most common TCM syndrome in DR [7].

Herb is an important part of TCM. Usually, herbs that were characterized with invigorating qi include Astragalus, yam, Atractylodes, and dangshen. Herbs that were characterized with enriching yin include raw radix rehmanniae, cooked rehmannia, wolfberry fruit, radix scrophulariae, dogwood, and fructus ligustri lucidi. Herbs that were characterized with blood activating include rhubarb, angelica, motherwort, red peony root, rhizoma ligustici wallichii, peach kernel, and cortexmoutan. Herbs that act to tonify qi and yin and invigorate blood (Yiqi Yangyin Huoxue) were beneficial for DR in many clinical trials. Studies of Yiqi Yangyin Huoxue method for DR have been conducted. However, owing to variation in the sample size and methodological quality of the studies, the efficacy of Yiqi Yangyin Huoxue method for DR is still not fully understood. Therefore, we conducted the present meta-analysis to review the efficacy of the Yiqi Yangyin Huoxue method for the treatment of DR.

## 2. Materials and Methods

### 2.1. Search Strategy

Six databases (PubMed, Medline, Cochrane Library, Weipu Journal, China Knowledge Network, and Wanfang database) were searched for patients before November 2018. The following domains of terms were used in combination, Yiqi Yangyin Huoxue, supplementing qi, nourishing yin, diabetic retinopathy, TCM, Chinese medicine, treat, and random. There was no restriction on language or study design. We also searched the bibliographies of retrieved articles for potentially relevant articles.

### 2.2. Including and Excluding Criteria

#### 2.2.1. Including Criteria

We included studies that met the following inclusion criteria: (1) types of studies: randomized controlled trials (RCTs); (2) type of participants: patients diagnosed with diabetic retinopathy either using 2002 American College of Ophthalmology severity scales for DR [8] or using the current classification criteria for DR in China [9]; (3) the main intervention which was Yiqi Yangyin Huoxue Chinese medicine, and the control group which was treated with Western medicine alone; at the same time both groups were given conventional treatment to control blood glucose; (4) outcomes which included the vision, eye fundus, FFA, and TCM syndromes.

#### 2.2.2. Excluding Criteria

We excluded trials that met the following exclusion criteria: (1) studies that were not RCTs; (2) the target population which was inconsistent with diagnostic criteria of DR; (3) Yiqi Yangyin Huoxue Chinese medicine which was used as an adjuvant treatment; (4) the study with duplicate publication; (5) reviews, letters, comments, and animal research; (6) low quality clinical trials.

### 2.3. Data Abstraction and Quality Assessment

Two reviewers independently retrieved the eligible studies according to the search strategy and selection criteria. Disagreement between two authors was resolved by discussion or consultation with a third reviewer. The extracted data included the first author(s), sample size, age, interventions details, outcomes, follow-up periods, and adverse events. Efficacy criteria of examination indicator referred to the guiding principles for clinical research of new Chinese medicine in 2002 [20]. Study quality was assessed by Cochrane Handbook 5.0.1 [21], which contains evaluation of randomization, allocation concealment, blinding, incomplete outcome data, withdrawals and dropouts, and other biases.

### 2.4. Statistical Analysis

Statistical analysis was performed using the Review Manager 5.3 software from the Cochrane Collaboration. In this meta-analysis, the mean difference (MD) and the risk ratio (RR) were used to assess continuous variable outcomes and dichotomous outcomes with a 95% confidence interval (CI). P<0.05 was considered statistically significant. A chi-square test with P value and the I^2^ statistic were used to quantify the statistical heterogeneity between studies. If no heterogeneity between studies was observed (P>0.1 or I^2^ <50%), the fixed effect model was used for the analysis; otherwise the random effect model was used. Forest plots displayed summary weighted estimates and the funnel plots could be used to assess the publication biases.

## 3. Results

### 3.1. Search Results

**Figure 1:**
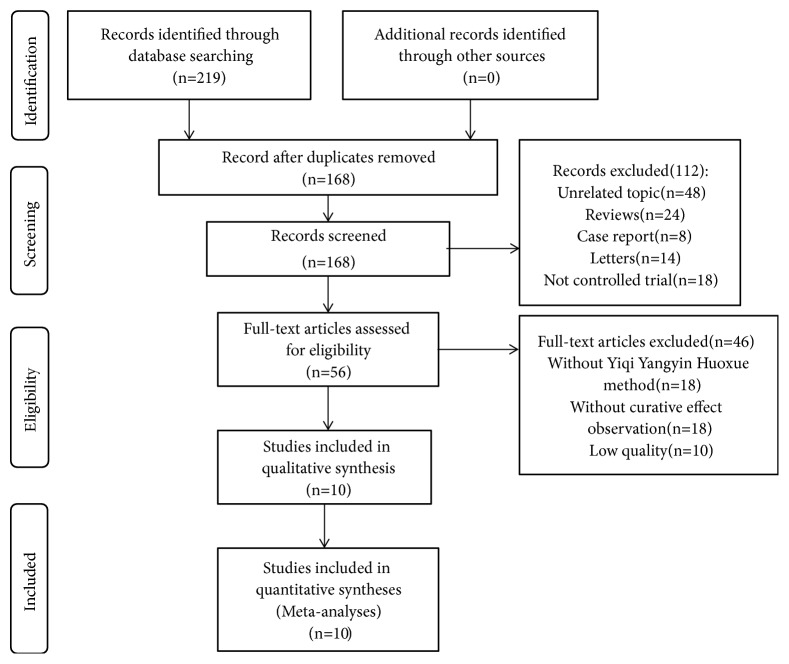
Flow diagram of included studies for this meta-analysis.

A total of 219 potential articles up to November 2018 were identified with the electronic-based search. After removing duplicates, 168 articles remained. We excluded 112 articles by screening titles and abstracts and retrieved the full texts of 56 remaining articles. Finally, 10 studies [10–19] met the inclusion criteria and were included in this meta-analysis (Figure 1).

### 3.2. Characteristics of the Eligible Studies

All of included studies were RCTs and the characteristics of these studies are summarized in Table 1. Studies were published from 2009 to 2018, all originated from China. The sample size of the 10 studies ranged from 34 to 122, and course of treatment varied from 2 to 52 weeks. The distribution of age and gender did not vary significantly in studies, and two studies report follow-up [10, 11].

### 3.3. Methodological Quality of Included Studies

All studies described a correct randomization procedure and incomplete outcome data, but none of them mentioned allocation concealment and blinding. Two out of 10 studies described withdrawals and dropouts [10, 14] (Table 2).

### 3.4. Treatment Effects

#### 3.4.1. Vision

**Figure 2:**
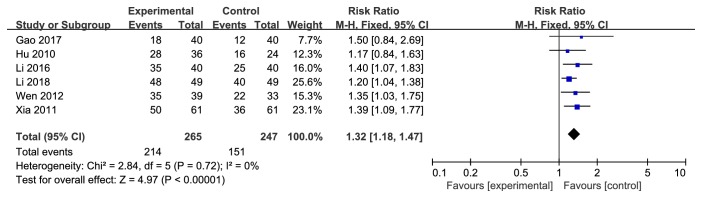
Forest plot showing the effect of Yiqi Yangyin Huoxue method for diabetic retinopathy in vision.

According to the vision changes in forest plot, the effect size of the 6 selected trials was RR=1.32 and 95% CI 1.18 to 1.47; test of the effect size was Z=4.97 and *p*<0.00001; and the heterogeneity analysis (*p*=0.72, I^2^=0%) suggests that there is no heterogeneity in 6 articles; the fixed effect model was used for the analysis. The aggregated results of 6 RCTs [10–15] suggested that Yiqi Yangyin Huoxue method showed favorable effects for improving vision of diabetic retinopathy (Figure 2).

#### 3.4.2. Eye Fundus

**Figure 3:**
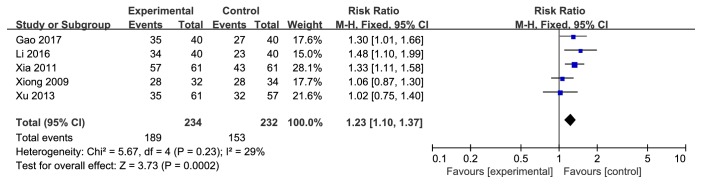
Forest plot showing the effect of Yiqi Yangyin Huoxue method for diabetic retinopathy in eye fundus.

According to the eye fundus changes in forest plot, the effect size of the 5 selected trials was RR=1.23 and 95% CI 1.10 to 1.37; test of the effect size was Z= 3.73 and *p*=0.0002; and the heterogeneity analysis (*p*=0.23, I^2^=29%) suggests that there is no heterogeneity in 5 articles; the fixed effect model was used for the analysis. The aggregated results of 5 studies [12, 13, 15, 18, 19] suggested that Yiqi Yangyin Huoxue method showed favorable effects for changing eye fundus of diabetic retinopathy while the other 2 trials [18, 19] failed to show this effect ( Figure 3).

#### 3.4.3. Fundus Fluorescence Angiography (FFA)

**Figure 4:**
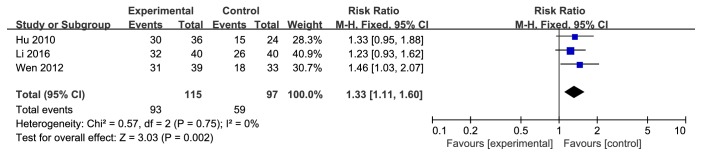
Forest plot showing the effect of Yiqi Yangyin Huoxue method for diabetic retinopathy in FFA.

According to the FFA changes in forest plot, the effect size of the 3 selected trials was RR=1.33 and 95% CI 1.11 to 1.60; test of the effect size was Z=3.03 and *p*=0.002; and the heterogeneity analysis (*p*=0.75, I^2^=0%) suggests that there is no heterogeneity in 3 articles; the fixed effect model was used for the analysis. The aggregated results of 3 studies [10–12] suggested that Yiqi Yangyin Huoxue method showed favorable effects for changing FFA of diabetic retinopathy (Figure 4). FFA showed that the original neovascularization subsided, no new neovascularization and vitreous hemorrhage, and no retinal capillary perfusion area disappeared.

#### 3.4.4. TCM Syndromes

**Figure 5:**
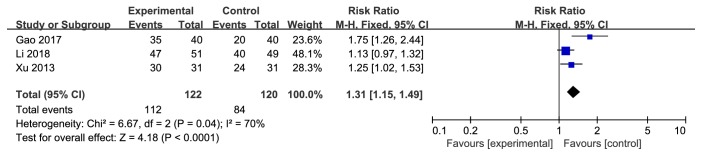
Forest plot showing the effect of Yiqi Yangyin Huoxue method for diabetic retinopathy in TCM syndromes.

According to TCM syndromes changes in forest plot, the effect size of the 3 selected trials was RR=1.31 and 95% CI 1.15 to 1.49; test of the effect size was Z=4.18 and *p* < 0.0001; and the heterogeneity analysis (*p*=0.04, I^2^=70%) suggests that there is heterogeneity in 3 articles. The aggregated results of 3 studies [14, 15, 18] suggested that Yiqi Yangyin Huoxue method showed favorable effects for changing TCM syndromes that the clinical symptoms and signs of TCM are significantly improved with the syndrome score greater than or equal to 70% (Figure 5).

#### 3.4.5. Fasting Blood Glucose (FBG)

**Figure 6:**
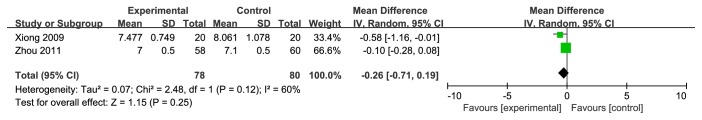
Forest plot showing the effect of Yiqi Yangyin Huoxue method for diabetic retinopathy in FBG.

According to FBG changes in forest plot, the effect size of the 2 selected trials was MD=-0.26 and 95% CI -0.71 to 0.19; test of the effect size was Z=1.15 and *p*=0.25 was not considered statistically significant. The aggregated results of 2 studies [16, 19] suggested that Yiqi Yangyin Huoxue method did not show favorable effects for decreasing FBG of diabetic retinopathy (Figure 6).

#### 3.4.6. Hemorheological Parameters

**Figure 7:**
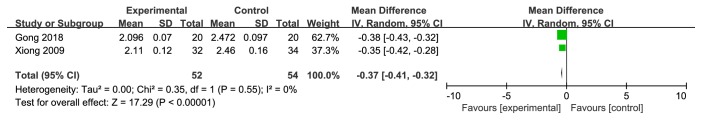
Forest plot showing the effect of Yiqi Yangyin Huoxue method for diabetic retinopathy in Hemorheological parameters.

The hemorheological parameters have blood viscosity, plasma viscosity, red cells metamorphosis, and red cells aggregation. The increased aggregation of red cells might be important account for blood pathological changes in diabetic retinopathy [22]. According to the red cells aggregation changes in forest plot, the effect size of the 2 selected trials was MD=-0.37 and 95% CI -0.41 to -0.32; test of the effect size was Z=17.29 and *p*<0.00001; and the heterogeneity analysis (*p*=0.55, I^2^=0%) suggests that there is no heterogeneity in 2 articles; the fixed effect model was used for the analysis. The aggregated results of 2 studies [17, 19] suggested that Yiqi Yangyin Huoxue method showed favorable effects for changing hemorheological parameters of diabetic retinopathy (Figure 7).

## 4. Discussion

This meta-analysis included 10 randomized clinical trials which applied to Yiqi Yangyin Huoxue method in the treatment of DR. All the trials have clear diagnostic criteria, inclusion criteria and exclusion criteria. Among the included 10 RCTs, 4 papers [10, 11, 13, 15] reported TMC with laser for treatment of proliferative diabetic retinopathy. The results showed that TCM and laser seem to be more effective in treatment of proliferative diabetic retinopathy by improving retinal microcirculation, increasing metabolization, and promoting the absorption of hemorrhage as supplement of laser. As indicated in this meta-analysis, Yiqi Yangyin Huoxue Method showed beneficial effects for DR on improving vision, eye fundus, FFA, TCM syndromes, and hemorheological parameters, but not for FBG. And there was no heterogeneity expecting TCM syndromes. Additionally, we did not apply funnel plot and Egger's test, because the small number of relevant studies had little power to correctly detect the risk of publication bias.

DR is one of the most common chronic complications of diabetes. The pathogenesis of DR is complex and not yet fully clarified. High blood glucose, activation of the protein kinase C, inflammation, oxidative stress, pigment epithelium-derived factor, and Epigenetic miRNA regulation contribute to the development of DR [23–26]. Western medicine in the treatment of DR mainly includes the strict control of blood glucose, retinal laser photocoagulation, intravitreal antivascular endothelial growth factor, and vitreous retinal surgery, but not exactly effective treatment with noninvasive. In TCM, the pathogenesis of DR is deficiency of qi and yin and qi failing to circulate blood. So the principle of treatment is to nourish qi and yin and promote blood circulation to resolve blood stasis. Each herbal product of Yiqi Yangyin Huoxue method within the TCM formulations could have several different active ingredients to attack a disease process. For example, Astragalus polysaccharide has prophylactic and therapeutic effects on the progress of DR by restraining protein kinase C and blocking pathway of PKC [27]. Rehmanniae could be applicable for the treatment of DR by improving function of pancreas islet cells, reducing resistance level of blood insulin, adjusting the balance of cellular glucose, and improving lipid metabolism disorder [28].

However, this meta-analysis was limited by the following factors: (1) The meta-analyses only included 10 studies assessing a total of 661 patients. (2) The included studies were not of very high quality and all were in China. (3) Most studies only mentioned the use of the random method, but the specific method used is unknown. (4) None of the study mentioned the allocation concealment and blinding. (5) Heterogeneity was inevitable due to the different traditional Chinese herbs in Yiqi Yangyin Huoxue method used. Based on the above limitations, it is difficult to draw a definite conclusion.

Therefore, in future clinical studies, we can carry out large-sample, high-quality, multicenter, multilevel, and properly blinded randomized controlled trials. Only by following the evidence-based medicine theory and conducting experiments under the unified standard strictly can improve the quality of meta-analysis. Thus, the meta-analysis has more guiding significance in clinical practice.

## 5. Conclusions

In conclusion, our systemic review initially demonstrated the therapeutic effects of Yiqi Yangyin Huoxue method in DR patients. Due to the limitation of this meta-analysis, more data in all follow-up phases and more RCTs should be required which can provide some guidance suggestions in the clinical.

## Figures and Tables

**Table 1:** Characteristics of the eligible studies.

Studies (first author, year)	Location	Patients No.	Age (mean years)	Genders (M/F)	Intervention group	Course of treatment (week)	Follow-up (mo)
Hu [10], 2010	China	34	57.6	18/16	TCM+ laser	2-6	3
Wen [11], 2012	China	46	50.5	24/22	TCM+ laser	2-6	6-12
Li [12], 2016	China	80	47.05	36/44	TCM	52	NMT
Xia [13], 2011	China	122	55	66/56	TCM+ laser	4	NMT
Li [14], 2018	China	98	57.89	43/55	TCM	8	NMT
Gao [15], 2017	China	80	55.5	36/44	TCM+ laser	12	NMT
Zhou [16], 2011	China	59	56	24/35	TCM	12	NMT
Gong [17], 2018	China	40	53.4	19/21	TCM	12	NMT
Xu [18], 2013	China	62	59.84	28/34	TCM	4	NMT
Xiong [19], 2009	China	40	57	19/21	TCM	12	NMT

NMT: not mentioned.

**Table 2:** Quality of the included studies.

Studies (first author, year)	Randomization	Allocation concealment	Blinding	Incomplete outcome data	Withdrawals and dropouts
Hu [10], 2010	Yes	NMT	NO	Yes	NMT
Wen [11], 2012	Yes	NMT	NO	Yes	NMT
Li [12], 2016	Yes	NMT	NO	Yes	MT
Xia [13], 2011	Yes	NMT	NO	Yes	NMT
Li [14], 2018	Yes	NMT	NO	Yes	NMT
Gao [15], 2017	Yes	NMT	NO	Yes	NMT
Zhou [16], 2011	Yes	NMT	NO	Yes	MT
Gong [17], 2018	Yes	NMT	NO	Yes	NMT
Xu [18], 2013	Yes	NMT	NO	Yes	NMT
Xiong [19], 2009	Yes	NMT	NO	Yes	NMT

MT: mentioned; NMT: not mentioned.
